# Quality of reporting of systematic reviews and meta‐analyses of surgical randomized clinical trials

**DOI:** 10.1002/bjs5.50266

**Published:** 2020-02-28

**Authors:** J. Yu, W. Chen, P. Wu, Y. Li

**Affiliations:** ^1^ Chinese Evidence‐based Medicine Centre, West China Hospital Sichuan University Chengdu China; ^2^ Editorial Office, West China Medical Press Sichuan University Chengdu China

## Abstract

**Background:**

Well designed and conducted systematic reviews are essential to clinical practice. Surgical intervention is more complex than medical intervention when considering special items related to procedures. There has been no cross‐sectional study of the reporting quality of systematic reviews of surgical randomized trials focused on special items relating to surgical interventions.

**Methods:**

A cross‐sectional survey of systematic reviews of surgical randomized trials published in 2007 and 2017 was undertaken via a PubMed search. Quality of reporting was assessed by the PRISMA checklist, with intervention details containing 27 items. Univariable and multivariable linear regression was used to explore factors in the checklist as indicators of reporting quality.

**Results:**

A total of 204 systematic reviews were identified. The median score for the PRISMA checklist was 22 (i.q.r. 20–24), and systematic reviews published in 2017 had a significantly higher median score than those from 2007 (22 (i.q.r. 21–24) *versus* 20 (17–22); *P* < 0·001). Among the 27 items, 15 were reported adequately and three were reported poorly (in less than 50 per cent of reports). The proportion of other items reported ranged from 54·4 to 77·9 per cent. In multivariable analysis, systematic reviews published in 2017 (coefficient 0·59, 95 per cent c.i. 0·50 to 0·69) and Cochrane reviews (coefficient 0·67, 0·55 to 0·81) were associated with better reporting.

**Conclusion:**

The quality of reporting of systematic reviews of surgical randomized trials has improved in the past 10 years. Some information relating to specific surgical interventions is, however, still reported poorly.

## Graphical abstract

Adherence to the PRISMA checklist was moderate; one‐half of the reporting items for systematic reviews (SRs) were reported adequately. The overall quality of reporting of SRs of surgical randomized trials improved between 2007 and 2017. These findings highlight the importance of describing the intervention in systematic reviews, especially for surgical research and complex interventions. A strong recommendation is made that adequate description of the intervention should be considered in SRs to improve the usability and reproducibility for review authors, peer reviewers and journal editors.

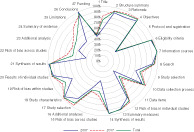

Still room for improvement

## Abstract (es)

**Antecedentes:**

En la práctica clínica es esencial disponer de revisiones sistemáticas bien diseñadas y realizadas. La intervención quirúrgica es más compleja que la intervención médica respecto a aspectos especiales relacionados con los procedimientos. No se ha realizado ningún estudio transversal de la calidad de las revisiones sistemáticas de ensayos aleatorizados quirúrgicos enfocado a evaluar aspectos especiales relacionados con las intervenciones quirúrgicas.

**Métodos:**

Se realizó una encuesta transversal de revisiones sistemáticas de ensayos quirúrgicos aleatorizados publicados en el 2007 o el 2017 mediante una búsqueda en PubMed. La calidad del informe se evaluó mediante la lista de verificación PRISMA que incluía 27 variables relacionadas con detalles de la intervención. Se realizó un análisis de regresión lineal univariable y multivariable para explorar qué factores de la lista de verificación era indicadores de la calidad de los informes.

**Resultados:**

Se identificaron 204 revisiones sistemáticas. La mediana (rango intercuartílico) de la puntuación PRISMA fue de 22 (20, 24), y las revisiones sistemáticas publicadas en el 2017 tuvieron una puntuación significativamente más alta que las de 2007 (22 (21, 24) *versus* 20 (17, 22) *P* < 0,001)). De las 27 variables, 15 se informaron adecuadamente y 3 se informaron mal en menos del 50% de las publicaciones. La proporción entre las otras variables varió entre el 55,4% y el 78,3%. En el análisis multivariable, las revisiones sistemáticas publicadas en el 2017 se asociaron con informes mejores (coeficiente 0,59, i.c. del 95% 0,50 a 0,69) y Cochrane (coeficiente 0,67, i.c. del 95% 0,55 a 0,81).

**Conclusión:**

La calidad de los informes de las revisiones sistemáticas de ensayos quirúrgicos aleatorizados ha mejorado en los últimos 10 años. Sin embargo, la información específica acerca de los detalles quirúrgicos todavía puede mejorar.

## Introduction

A systematic review (SR) aims to collate all empirical evidence that fits prespecified eligibility criteria to answer a specific research question^1^. Explicit SR methods are available to identify gaps in current research, minimize bias, and provide reliable and timely conclusions^2^, ^3^. SRs have become essential to clinical guideline development and health policy decision‐making^4^, ^5^. A SR, however, may be of limited use in clinical practice if reporting is incomplete^6^, ^7^.

To improve the completeness in reporting of SRs and meta‐analyses, the Quality Of Reporting Of Meta‐analyses (QUOROM) statement^8^ was developed in 1999, followed in 2009 by the PRISMA statement^9^. The PRISMA statement is a reporting guideline designed to offer a standard approach for review authors to prepare and report a SR transparently and reproducibly. Having been developed originally to aggregate outcomes from therapeutic trials, extended versions of PRISMA have been developed in relation to diagnostic test accuracy reviews^10^, harms^11^, protocol^12^, equity^13^, network meta‐analyses^14^ and complex interventions^15^.

Surgical intervention is more complex than simple comparisons between drugs. Special items are needed, related to surgical technology, devices, quality control measures related to the surgical procedures, and the surgeons' experience, for example.

There has been no cross‐sectional study of the reporting quality of SRs of surgical RCTs with special items relating to surgical interventions. The present study was undertaken as a cross‐sectional survey to investigate the extent to which SRs of surgical randomized studies adhered to the modified PRISMA checklist that combined items with intervention details. It was also investigated whether the quality of reporting had improved by comparing 2007 and 2017, with consideration of factors associated with the quality of SRs.

## Methods

A study was included if it was a SR with meta‐analysis of randomized trials published in 2007 or 2017, in English, that compared a surgical intervention with other interventions (another surgical intervention or a non‐surgical treatment). A SR was defined as described in the Cochrane handbook (version 5.1.0)^1^, as an article with clearly stated objectives with an explicitly reproducible methodology, systematic search methods, assessment of the validity of included studies and methods of synthesis. The definition of a surgical intervention has been described elsewhere^16^.

Narrative and other types of review were excluded along with systematic reviews that included crossover trials, individual participant data (IPD) meta‐analyses and network meta‐analyses. Studies testing injection, acupuncture or diagnostic interventions were not eligible, and those that tested the effects of medical therapies in patients undergoing surgical procedures, such as antibiotics or adjuvant treatments in cancer, were also excluded.

### Data resource and study procedure

Studies published in 2007 and 2017 were identified via PubMed. The search strategy was based on MeSH (Medical Subject Heading) terms and their variants (*Appendix* *S1*, supporting information). Two reviewers, trained in trial and SR methods, screened abstracts and full texts for eligibility, and abstracted data from eligible studies with detailed introductions. Disagreement was resolved by a third reviewer if necessary.

### Data collection

The following general information was collected from each eligible study: number of authors; number of studies included in the SR; total number of participants included; number of databases searched; type of review (Cochrane or non‐Cochrane); reference to PRISMA or QUOROM statement; involvement of a methodologist (such as a statistician or epidemiologist); country; journal type (general or surgical journal); type of comparison; specialty; type of funding (profit, non‐profit, not funded or not reported); and significance of the primary outcome (yes, no or unclear). A primary outcome was selected according to published studies^17^: if a SR specified a primary outcome, this was chosen as the primary outcome in the present analysis. Where more than one primary outcome existed, the first one reported in methods was selected. If no primary outcome was specified, the first outcome reported in the abstract was chosen.

The items in the PRISMA checklist were used to assess reporting quality. The checklist consists of 27 items, and quality of reporting was assessed against these 27 items for each study. One point was allocated if the study met the requirement for a specific item, and no points if it did not. Each item was determined with the option of ‘yes’ or ‘no’. Given that some items of information, such as surgical technique, preoperative care, postoperative care, rehabilitation protocol, devices and surgeon experience, are important to the application of SRs, these specific items were considered under the headings of ‘data items’ and ‘summary of evidence’ (*Appendix* *S2*, supporting information). A score ranging from 0 to 27 was thus developed.

### Statistical analysis

Proportions were calculated for categorical variables, and mean(s.d.), median (range) or median (i.q.r.) values for continuous variables. Either χ^2^ or Fisher's exact tests were used for analysis of categorical variables. The Wilcoxon rank sum test was employed to analyse continuous data with a non‐normal distribution. Univariable and multivariable linear regression analyses were used to assess the association between the six variables specified by the PRISMA statement (27 items). The six factors included: year of publication (2017 *versus* 2007); type of systematic review (Cochrane *versus* non‐Cochrane); type of journal (surgical *versus* general); author with methodological affiliation (yes *versus* no); significance of primary outcome (yes *versus* no/unclear); and source of funding (non‐profit *versus* profit; none/unclear *versus* profit). Skewness and kurtosis tests were used to check the distribution of scores^18^. The variance inflation factor was used to explore for multicollinearity among studies^19^.

SPSS® version 22.0 (IBM, Armonk, New York, USA) was used to conduct the statistical analysis.

## Results

**Figure 1:**
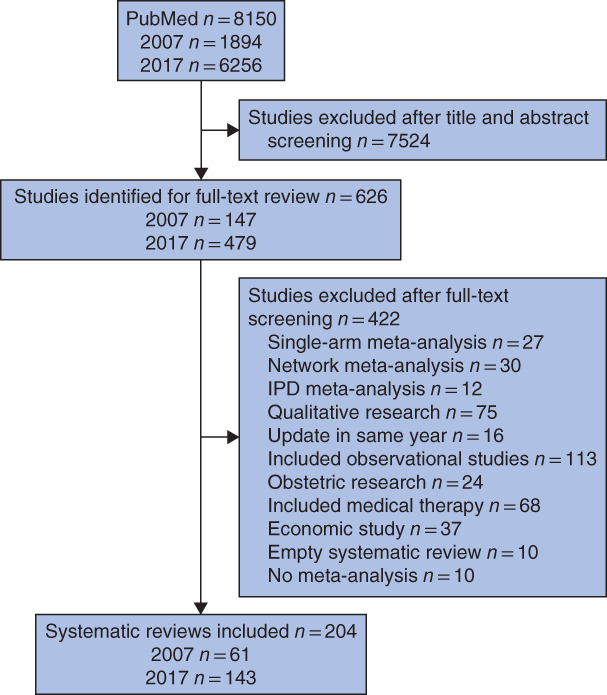
Study selectionIPD, individual participant data.

A total of 8150 studies were identified. After title and abstract screening, 626 studies were potentially eligible. On full‐text screening, 204 systematic reviews were finally included (*Fig*. 1). References in the included studies are shown in *Appendix* *S3* (supporting information).

The general characteristics of the included SRs are shown in *Table* 1. The median number of participants included was 1149 (range 55–27 807) and the median number of databases involved was 4 (1–15). Some 32 mentioned authors (15·7 per cent) were affiliated to a department of epidemiology, statistics or evidence‐based medicine, or a methodologist was listed in the acknowledgements section. Some 43 SRs (21·1 per cent) were Cochrane reviews, 61 (29·9 per cent) were published in surgical journals, 177 (86·8 per cent) compared alternative surgical interventions, 56 (27·5 per cent) reported the funding source and 43 (21·1 per cent) received no funding. Studies published in 2017 included more authors than SRs published in 2007 (median 6 *versus* 4 respectively; *P* < 0·001), although they included fewer studies (7 *versus* 10; *P* = 0·013). Fewer Cochrane SRs were found in 2017 (16·8 *versus* 31 per cent; *P* = 0·021), although the number of SRs of surgical RCTs had increased in some countries, notably China. SRs from 2017 were more likely to declare clearly that they followed the reporting standards of the PRISMA or QUOROM checklist (57·3 per cent *versus* 10 per cent in 2007; *P* < 0·001).

**Table 1:** Characteristics of included systematic reviews

	Total (*n* = 204)	2007 (*n* = 61)	2017 (*n* = 143)	*P* †
**No. of authors** *	5 (2–21)	4 (2–20)	6 (2–21)	< 0·001‡
**No. of studies included** *	7 (1–44)	10 (1–42)	7 (1–44)	0·013‡
**No. of participants included** *	1149 (55–27 807)	1148 (55–27 807)	1167 (80–19 886)	0·664‡
**No. of databases included** *	4 (1–15)	4 (1–7)	4 (1–15)	0·126‡
**Methodologist involved**	32 (15·7)	14 (23)	18 (12·6)	0·062
**Cochrane SR**	43 (21·1)	19 (31)	24 (16·8)	0·021
**PRISMA/QUOROM mentioned**	88 (43·1)	6 (10)	82 (57·3)	< 0·001
**Type of journal**				0·679
General	143 (70·1)	44 (72)	99 (69·2)	
Surgical	61 (29·9)	17 (28)	44 (30·8)	
**Country of corresponding author**				< 0·001§
China	48 (23·5)	2 (3)	46 (32·2)	
UK	33 (16·2)	19 (31)	14 (9·8)	
USA	31 (15·2)	11 (18)	20 (14·0)	
Australia	14 (6·9)	4 (7)	10 (7·0)	
Italy	15 (7·4)	4 (7)	11 (7·7)	
Other	63 (30·9)	21 (34)	42 (29·4)	
**Type of comparison**				0·303§
Surgical *versus* surgical	177 (86·8)	51 (84)	126 (88·1)	
Surgical *versus* non‐surgical	22 (10·8)	7 (11)	15 (10·5)	
Surgical *versus* both	5 (2·5)	3 (5)	2 (1·4)	
**Specialty**				0·914§
General surgical	72 (35·3)	21 (34)	51 (35·7)	
Cardiothoracic	60 (29·4)	17 (28)	43 (30·1)	
Orthopaedic	31 (15·2)	9 (15)	22 (15·4)	
Urological	12 (5·9)	5 (8)	7 (4·9)	
Other	29 (14·2)	9 (15)	20 (14·0)	
**Funding**				0·645§
Profit	3 (1·5)	0 (0)	3 (2·1)	
Non‐profit	53 (26·0)	18 (30)	35 (24·5)	
Not funded	43 (21·1)	10 (16)	33 (23·1)	
Funding not reported	105 (51·5)	33 (54)	72 (50·3)	

Values in parentheses are percentages unless indicated otherwise;

* values are median (range). SR, systematic review; QUOROM, Quality Of Reporting Of Meta‐analyses.

† Pearson's χ^2^ test, except

‡ Mann–Whitney *U* test and §Fisher's exact test.

**Figure 2:**
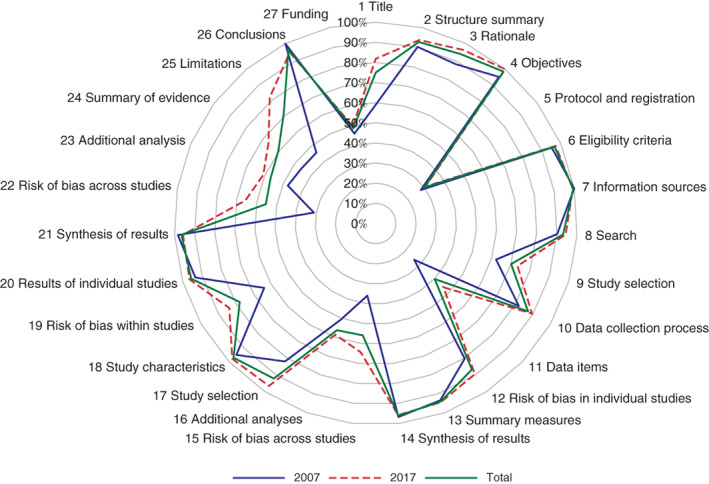
Proportion of studies adhering to the PRISMA checklist

*Table* 2 and *Fig*. 2 show adherence to the PRISMA checklist. Interobserver agreement on PRISMA was good (κ statistic = 0·78). Of the 27 items, 15 (56 per cent) were reported adequately. Three items were reported less than 50 per cent of the time: accessibility of a review protocol and registration information (29·9 per cent); statement of all variables where data was sought, especially the description of intervention details (40·2 per cent); and description of funding source for SRs (48·5 per cent).

**Table 2:** Adherence to systematic review reporting

Section	Items	Total (*n* = 204)	2007 (*n* = 61)	2017 (*n* = 143)	*P* †
Title	1 Title	153 (75·0)	36 (59)	117 (81·8)	< 0·001
Abstract	2 Structure summary	189 (92·6)	55 (90)	134 (93·7)	0·390
Introduction	3 Rationale	192 (94·1)	54 (89)	138 (96·5)	0·046‡
	4 Objectives	201 (98·5)	58 (95)	143 (100)	0·026‡
Methods	5 Protocol and registration	61 (29·9)	17 (28)	44 (30·8)	0·680
	6 Eligibility criteria	197 (96·6)	58 (95)	139 (97·2)	0·430‡
	7 Information sources	203 (99·5)	61 (100)	142 (99·3)	1·000‡
	8 Search	190 (93·1)	55 (90)	135 (94·4)	0·360
	9 Study selection	143 (70·1)	38 (62)	105 (73·4)	0·110
	10 Data collection process	178 (87·3)	50 (82)	128 (89·5)	0·140
	11 Data items	82 (40·2)	16 (26)	66 (46·2)	0·008
	12 Risk of bias in individual studies	177 (86·8)	49 (80)	128 (89·5)	0·076
	13 Summary measures	192 (94·1)	57 (93)	135 (94·4)	0·750‡
	14 Synthesis of results	196 (96·1)	59 (97)	137 (95·8)	1·000‡
	15 Risk of bias across studies	114 (55·9)	22 (36)	92 (64·3)	< 0·001
	16 Additional analyses	115 (56·4)	31 (51)	84 (58·7)	0·300
Results	17 Study selection	188 (92·2)	50 (82)	138 (96·5)	0·001‡
	18 Study characteristics	198 (97·1)	58 (95)	140 (97·9)	0·370‡
	19 Risk of bias within studies	159 (77·9)	39 (64)	120 (83·9)	0·003
	20 Results of individual studies	195 (95·6)	57 (93)	138 (96·5)	0·460‡
	21 Synthesis of results	197 (96·6)	60 (98)	137 (95·8)	1·000‡
	22 Risk of bias across studies	113 (54·4)	19 (31)	94 (65·7)	< 0·001
	23 Additional analysis	116 (56·9)	29 (48)	87 (60·8)	0·080
Discussion	24 Summary of evidence	123 (60·3)	28 (46)	95 (66·4)	0·006‡
	25 Limitations	145 (71·1)	28 (46)	117 (81·8)	< 0·001
	26 Conclusions	197 (96·6)	61 (100)	136 (95·1)	0·110‡
Funding	27 Funding	99 (48·5)	28 (46)	71 (49·7)	0·620
Summarized scores	PRISMA*	22 (20–24)	20 (17–22)	22 (21–24)	< 0·001§

Values in parentheses are percentages unless indicated otherwise

* values are median (i.q.r.).

† Pearson's χ^2^ test, except

‡ Fisher's exact test

§ Mann–Whitney *U* test.

The median score for the PRISMA checklist was 22 (i.q.r. 20–24), and SRs published in 2017 had a significantly higher score than those from 2007 (22 (21–24) *versus* 20 (17–22); *P* < 0·001). SRs published in 2017 were more likely than those from 2007 to report the title (81·8 *versus* 59 per cent respectively; *P* < 0·001), objective (100 *versus* 95 per cent; *P* = 0·026), data items (46·2 *versus* 26 per cent; *P* = 0·008), method used to assess the risk of bias across studies (64·3 *versus* 36 per cent; *P* < 0·001), study selection (96·5 *versus* 82 per cent; *P* = 0·001), results of risk of bias within studies (83·9 *versus* 64 per cent; *P* = 0·003) and across studies (65·7 *versus* 31 per cent; *P* < 0·001), summary of evidence (66·4 *versus* 46 per cent; *P* = 0·006) and limitations (81·8 *versus* 46 per cent; *P* < 0·001).

Univariable analysis showed that more recent publications (2017 *versus* 2007), Cochrane SRs, and studies published in general journals were associated with a higher score (*Table* 3). In multivariable analysis, SRs published in 2017 (coefficient 0·59, 95 per cent c.i. 0·50 to 0·69) and Cochrane methodology (coefficient 0·67, 0·55 to 0·81) were associated with better reporting.

**Table 3:** Univariable and multivariable linear regression analysis of factors associated with quality of reporting (PRISMA checklist)

	Univariable analysis		Multivariable analysis	
	Coefficient	*P*	Coefficient	*P*
**Year of publication (2017 *versus* 2007)**	0·63 (0·53, 0·74)	< 0·001	0·59 (0·50, 0·69)	< 0·001
**Systematic review type (Cochrane *versus* non‐Cochrane)**	0·71 (0·58, 0·86)	< 0·001	0·67 (0·55, 0·81)	< 0·001
**Journal type (surgical *versus* general)**	1·30 (1·09, 1·55)	0·003	1·19 (1·01, 1·40)	0·042
**Author with statistical or epidemiological affiliation (yes *versus* no)**	0·99 (0·1, 1·24)	0·937	1·08 (0·89, 1·32)	0·429
**Significance of primary outcome (yes *versus* no/unclear)**	0·67 (0·79, 3·39)	0·624	0·95 (0·82, 1·11)	0·517
**Source of funding**				
Non‐profit *versus* profit	1·07 (0·33, 1·28)	0·476	0·77 (0·42, 1·41)	0·397
None/unclear *versus* profit	0·92 (0·77, 1·10)	0·354	0·70 (0·38, 1·26)	0·232

Values in parentheses are 95 per cent confidence intervals.

## Discussion

This cross‐sectional survey of 204 SRs of surgical randomized trials has demonstrated that compliance with the PRISMA checklist was moderate. Only half of the reporting items for SRs were reported adequately. Some items, such as registration, adequate description of intervention and funding, were missing regularly from many studies. The reporting of protocols and registration in SRs is much lower than in randomized trials^16^, ^20^, ^21^, ^22^, ^23^, ^24^, ^25^, reflecting compulsory requirement that a clinical trial must be registered before publication by the International Committee of Medical Journal Editors^26^.

Surgical intervention details, such as devices, nursing care, anaesthetic management and surgeon experience that may be critical to the inferences drawn from SRs, especially SRs with surgery‐related primary outcomes, were not well reported. Only 40·2 per cent of SRs included an adequate description of surgical interventions, and just 60·3 per cent discussed the effect of interventions on main outcomes. This places significant limitations on the value of SRs relating to these trials. An expert consensus^27^ to improve the consideration and description of interventions in SRs was published in 2017. It recommended that authors of SRs should consider intervention details when planning, conducting and reporting SRs, and suggested that review authors should provide a table summarizing the intervention details for each study^28^.

A limited number of SRs reported additional analyses (such as subgroup analyses or meta‐regression), but heterogeneity, led by clinical and methodological diversity, existed in all reviews, as noted elsewhere^29^, ^30^. Review authors should consider the feasibility and appropriateness of subgroup analyses or meta‐regression at the time of planning.

Despite being reported inadequately, the overall quality of reporting of SRs improved between 2007 and 2017. This improvement may be explained by the wide adoption of the PRISMA statement, increased awareness of PRISMA, and instructions from scientific journals regarding manuscripts relating to SRs^31^, ^32^. Completeness of reporting was superior from Cochrane compared with non‐Cochrane SRs, possibly owing to rigorous strategies in the editorial process and the absence of word limits^33^. The results of the present study are similar to those that included a wider range of study designs^34^, ^35^, ^36^.

There are several strengths to the present study. A validated effective search filter was used to identify all SRs published in 2007 and 2017 in PubMed, leading to greater generalizability of the findings. Rigorous methods, including explicit eligibility criteria, double‐checking of study screening and data extraction, were used. The study also has limitations. Authors' affiliation to a methodological department was determined only by the information provided on the title and acknowledgement pages. This approach may not have captured fully the expertise among authors regarding epidemiology or statistics. Items were reported as included or absent, with no qualitative assessment. The exclusion of narrative SRs, rapid reviews, overviews of reviews, and SRs with IPD meta‐analyses or network meta‐analyses means that the present results should not be considered generalizable to these other study designs. The present study also considered only SRs published in English.

Despite these limitations, the quality of reporting in SRs of surgical randomized trials has improved in the past 10 years. The overall findings, however, make a strong case, in particular, for the description of interventions to improve the usefulness of surgical SRs.

## Supporting information


**Appendix S1** Search strategy
**Appendix S2** PRISMA checklist with extension for intervention
**Appendix S3** References in included studies
