# Supplementary material for: Quality of reporting of systematic reviews and meta‐analyses of surgical randomized clinical trials
Source: BJS Open. 2020 Feb 28;4(3):535–42. doi: 10.1002/bjs5.50266 (PMC7260405; doi:10.1002/bjs5.50266)
Supplement: Supplementary file 1 — Appendix S1 Search strategy Appendix S2 PRISMA checklist with extension for intervention Appendix S3 References in included studies [file BJS5-4-535-s001.docx]

**BJS5_50266**

**Quality of reporting of systematic reviews and meta-analyses of surgical randomized clinical trials**

**J. Yu, W. Chen, P. Wu and Y. Li**

**Appendix S1** Search strategy

**PubMed:**

#1 "Specialties, Surgical"[Mesh]

#2 "Surgical Procedures, Operative"[Mesh]

#3 "Surgery" [Subheading]

#4 Surgery [tw] OR surgeries [tw] OR surgerys [tw]

#5 Operation [tw] OR operations [tw]

#6 Surgical procedure*[tw] OR Surgical operation*[tw] OR Surgical treatment*[tw] OR Surgical therap*[tw] OR Surgical intervention*[tw] OR Surgical research*[tw]

#7 Operative procedure*[tw] OR Operative operation*[tw] OR Operative treatment*[tw] OR Operative therap*[tw] OR Operative intervention*[tw] OR Operative research*[tw] Invasive procedure*[tw] OR Invasive intervention*[tw]

#8 Peroperative procedure*[tw] OR Peroperative intervention*[tw] OR Perioperative procedure*[tw] OR Perioperative intervention*[tw] OR Preoperative procedure*[tw] OR Preoperative intervention*[tw] OR Intraoperative procedure*[tw] OR Intraoperative intervention*[tw]

#8 #1 OR #2 OR #3 OR #4 OR #5 OR #6 OR #7

#9 "Review Literature as Topic"[Mesh] OR "Meta-Analysis as Topic"[Mesh]

#10 "Meta-Analysis" [Publication Type]

#11 Meta-analy*[tw] OR meta analy*[tw] OR Metaanaly*[tw] OR systematic review*[tw]

#12 Cochrane[tiab] OR Embase[tiab] OR medline[tiab] OR pubmed[tiab] OR medlars[tiab]

#13 Psychlit[tiab] OR psyclit[tiab] OR Cinahl[tiab] OR cinhal[tiab] OR Science citation index[tiab] OR Bids[tiab] OR Cancerlit[tiab]

#14 Reference list*[tiab] OR bibliograph*[tiab] OR Hand-search*[tiab] OR Relevant journals[tiab] OR Manual search*[tiab]

#15 Selection criteria[tiab] OR data extraction[tiab]) AND Review[Publication Type]

#16 Comment[Publication Type] OR letter[Publication Type] OR editorial[Publication Type]

#17 #9 OR #10 OR #11 OR #12 OR #13 OR #14 OR #15 OR #16

#18 #8 AND #17

#19 animals [Mesh]

#20 humans [Mesh]

#21 #19 NOT #20

#22 #18 NOT #21

**Appendix S2** PRISMA checklist with extension for intervention

| **Section/topic** | **Items** | **Checklist item** | **Extension for intervention** |
| --- | --- | --- | --- |
| **TITLE** | | | |
| Title | 1 | Identify the report as a systematic review, meta-analysis, or both. |  |
| **ABSTRACT** | | | |
| Structured summary | 2 | Provide a structured summary including, as applicable: background; objectives; data sources; study eligibility criteria, participants, and interventions; study appraisal and synthesis methods; results; limitations; conclusions and implications of key findings; systematic review registration number. |  |
| **INTRODUCTION** | | | |
| Rationale | 3 | Describe the rationale for the review in the context of what is already known. |  |
| Objectives | 4 | Provide an explicit statement of questions being addressed with reference to participants, interventions, comparisons, outcomes, and study design (PICOS). |  |
| **METHODS** | | | |
| Protocol and registration | 5 | Indicate if a review protocol exists, if and where it can be accessed (e.g., Web address), and, if available, provide registration information including registration number. |  |
| Eligibility criteria | 6 | Specify study characteristics (e.g., PICOS, length of follow-up) and report characteristics (e.g., years considered, language, publication status) used as criteria for eligibility, giving rationale. | describe the intervention details when applicable |
| Information sources | 7 | Describe all information sources (e.g., databases with dates of coverage, contact with study authors to identify additional studies) in the search and date last searched. |  |
| Search | 8 | Present full electronic search strategy for at least one database, including any limits used, such that it could be repeated. |  |
| Study selection | 9 | State the process for selecting studies (i.e., screening, eligibility, included in systematic review, and, if applicable, included in the meta-analysis). |  |
| Data collection process | 10 | Describe method of data extraction from reports (e.g., piloted forms, independently, in duplicate) and any processes for obtaining and confirming data from investigators. |  |
| Data items | 11 | List and define all variables for which data were sought (e.g., PICOS, funding sources) and any assumptions and simplifications made. | Surgery-related information: surgical technique, preoperative care, postoperative care, rehabilitation protocol, devices, surgeons’ experience, anesthetic management |
| Risk of bias in individual studies | 12 | Describe methods used for assessing risk of bias of individual studies (including specification of whether this was done at the study or outcome level), and how this information is to be used in any data synthesis. |  |
| Summary measures | 13 | State the principal summary measures (e.g., risk ratio, difference in means). |  |
| Synthesis of results | 14 | Describe the methods of handling data and combining results of studies, if done, including measures of consistency (e.g., I^2^) for each meta-analysis. |  |
| Risk of bias across studies | 15 | Specify any assessment of risk of bias that may affect the cumulative evidence (e.g., publication bias, selective reporting within studies). |  |
| Additional analyses | 16 | Describe methods of additional analyses (e.g., sensitivity or subgroup analyses, meta-regression), if done, indicating which were pre-specified. |  |
| **RESULTS** | | | |
| Study selection | 17 | Give numbers of studies screened, assessed for eligibility, and included in the review, with reasons for exclusions at each stage, ideally with a flow diagram. |  |
| Study characteristics | 18 | For each study, present characteristics for which data were extracted (e.g., study size, PICOS, follow-up period) and provide the citations. |  |
| Risk of bias within studies | 19 | Present data on risk of bias of each study and, if available, any outcome level assessment (see item 12). |  |
| Results of individual studies | 20 | For all outcomes considered (benefits or harms), present, for each study: (a) simple summary data for each intervention group (b) effect estimates and confidence intervals, ideally with a forest plot. |  |
| Synthesis of results | 21 | Present results of each meta-analysis done, including confidence intervals and measures of consistency. |  |
| Risk of bias across studies | 22 | Present results of any assessment of risk of bias across studies (see Item 15). |  |
| Additional analysis | 23 | Give results of additional analyses, if done (e.g., sensitivity or subgroup analyses, meta-regression [see Item 16]). |  |
| **DISCUSSION** | | | |
| Summary of evidence | 24 | Summarize the main findings including the strength of evidence for each main outcome; consider their relevance to key groups (e.g., healthcare providers, users, and policy makers). | Summarize the main findings including the effect of intervention for main outcome and describe implication for future research |
| Limitations | 25 | Discuss limitations at study and outcome level (e.g., risk of bias), and at review-level (e.g., incomplete retrieval of identified research, reporting bias). |  |
| Conclusions | 26 | Provide a general interpretation of the results in the context of other evidence, and implications for future research. |  |
| **FUNDING** | | | |
| Funding | 27 | Describe sources of funding for the systematic review and other support (e.g., supply of data); role of funders for the systematic review. |  |

**Appendix S3** References in included studies

1. Bainbridge D, Cheng D, Martin J, Novick R. Does off-pump or minimally invasive coronary artery bypass reduce mortality, morbidity, and resource utilization when compared with percutaneous coronary intervention? A meta-analysis of randomized trials. J Thorac Cardiov Sur 2007; 133(3):623-31.
2. Bath-Hextall FJ, Perkins W, Bong J, Williams HC. Interventions for basal cell carcinoma of the skin. Cochrane Database Syst Rev 2007(1):CD003412.
3. Bennett J, Boddy A, Rhodes M. Choice of approach for appendicectomy: a meta-analysis of open versus laparoscopic appendicectomy. Surg Laparosc Endosc Percutan Tech 2007; 17(4):245-55.
4. Biau DJ, Tournoux C, Katsahian S, Schranz P, Nizard R. ACL reconstruction: a meta-analysis of functional scores. Clin Orthop Relat Res 2007; 458(5):180-7.
5. Boyden TF, Nallamothu BK, Moscucci M, Chan PS, Grossman PM, Tsai TT, et al. Meta-analysis of randomized trials of drug-eluting stents versus bare metal stents in patients with diabetes mellitus. Am J Cardiol 2007; 99(10):1399-402.
6. Bradley DJ, Shen WK. Atrioventricular junction ablation combined with either right ventricular pacing or cardiac resynchronization therapy for atrial fibrillation: the need for large-scale randomized trials. Heart Rhythm 2007; 4(2):224-32.
7. Bravata DM, Gienger AL, McDonald KM, Sundaram V, Perez MV, Varghese R, et al. Systematic review: the comparative effectiveness of percutaneous coronary interventions and coronary artery bypass graft surgery. Ann Intern Med 2007; 147(10):703-16.
8. Brouwer RW, Raaij van TM, Bierma-Zeinstra SM, Verhagen AP, Jakma TS, Verhaar JA. Osteotomy for treating knee osteoarthritis. Cochrane Database Syst Rev 2007(3):CD004019.
9. Burzotta F, Testa L, Giannico F, Biondi-Zoccai GG, Trani C, Romagnoli E, et al. Adjunctive devices in primary or rescue PCI: a meta-analysis of randomized trials. Int J Cardiol 2007; 123(3):313-21.
10. Choy PY, Bissett IP, Docherty JG, Parry BR, Merrie AE. Stapled versus handsewn methods for ileocolic anastomoses. Cochrane Database Syst Rev 2007(3):CD004320.
11. De Luca G, Suryapranata H, Stone GW, Antoniucci D, Biondi-Zoccai G, Kastrati A, et al. Coronary stenting versus balloon angioplasty for acute myocardial infarction: a meta-regression analysis of randomized trials. Int J Cardiol 2007; 126(1):37-44.
12. De Luca G, Suryapranata H, Stone GW, Antoniucci D, Neumann FJ, Chiariello M. Adjunctive mechanical devices to prevent distal embolization in patients undergoing mechanical revascularization for acute myocardial infarction: a meta-analysis of randomized trials. Am Heart J 2007; 153(3):343-53.
13. Diener MK, Knaebel HP, Heukaufer C, Antes G, Buchler MW, Seiler CM. A systematic review and meta-analysis of pylorus-preserving versus classical pancreaticoduodenectomy for surgical treatment of periampullary and pancreatic carcinoma. Ann Surg 2007; 245(2):187-200.
14. Ederle J, Featherstone RL, Brown MM. Percutaneous transluminal angioplasty and stenting for carotid artery stenosis. Cochrane Database Syst Rev 2007(4):CD000515.
15. Field ML, Rengarajan A, Khan O, Spyt T, Richens D. Preoperative intra aortic balloon pumps in patients undergoing coronary artery bypass grafting. Cochrane Database Syst Rev 2007(1):CD004472.
16. Gibson JN, Waddell G. Surgical interventions for lumbar disc prolapse. Cochrane Database Syst Rev 2007(2):CD001350.
17. Gluud LL, Klingenberg S, Nikolova D, Gluud C. Banding ligation Versus beta-blockers as primary prophylaxis in esophageal varices: Systematic review of randomized trials. Am J Gastroenterol 2007; 102(12):2842-48.
18. Gurm HS, Nallamothu BK, Yadav J. Safety of carotid artery stenting for symptomatic carotid artery disease: a meta-analysis. Eur Heart J 2007; 29(1):113-9.
19. Gurusamy KS, Samraj K. Routine abdominal drainage for uncomplicated open cholecystectomy. Cochrane Database Syst Rev 2007(2):CD006003.
20. Gurusamy KS, Samraj K. Primary closure versus T-tube drainage after laparoscopic common bile duct stone exploration. Cochrane Database Syst Rev 2007(1):CD005641.
21. Gurusamy KS, Samraj K. Primary closure versus T-tube drainage after open common bile duct exploration. Cochrane Database Syst Rev 2007(1):CD005640.
22. Gurusamy KS, Samraj K, Davidson BR. Routine abdominal drainage for uncomplicated liver resection. Cochrane Database Syst Rev 2007(3):CD006232.
23. Gurusamy KS, Samraj K, Mullerat P, Davidson BR. Routine abdominal drainage for uncomplicated laparoscopic cholecystectomy. Cochrane Database Syst Rev 2007(4):CD006032.
24. Handoll HH, Huntley JS, Madhok R. External fixation versus conservative treatment for distal radial fractures in adults. Cochrane Database Syst Rev 2007(3):CD006194.
25. Handoll HH, Vaghela MV, Madhok R. Percutaneous pinning for treating distal radial fractures in adults. Cochrane Database Syst Rev 2007(3):CD006080.
26. Ho YH, Buettner PG. Open compared with closed haemorrhoidectomy: meta-analysis of randomized controlled trials. Tech Coloproctol 2007; 11(2):135-43.
27. Hodson EM, Wheeler DM, Vimalchandra D, Smith GH, Craig JC. Interventions for primary vesicoureteric reflux. Cochrane Database Syst Rev 2007(3):CD001532.
28. Hosono S, Osaka H. Minilaparoscopic versus conventional laparoscopic cholecystectomy: a meta-analysis of randomized controlled trials. J Laparoendosc Adv Surg Tech A 2007; 17(2):191-9.
29. Jackson TD, Kaplan GG, Arena G, Page JH, Rogers SO, Jr. Laparoscopic versus open resection for colorectal cancer: a metaanalysis of oncologic outcomes. J Am Coll Surg 2007; 204(3):439-46.
30. Jaffery Z, Kowalski M, Weaver WD, Khanal S. A meta-analysis of randomized control trials comparing minimally invasive direct coronary bypass grafting versus percutaneous coronary intervention for stenosis of the proximal left anterior descending artery. Eur J Cardio-Thorac 2007; 31(4):691-97.
31. Jayaraman S, Colquhoun PHD, Malthaner RA. Stapled hemorrhoidopexy is associated with a higher long-term recurrence rate of internal hemorrhoids compared with conventional excisional hemorrhoid surgery. Dis Colon Rectum 2007; 50(9):1297-305.
32. Kahnamoui K, Cadeddu M, Farrokhyar F, Anvari M. Laparoscopic surgery for colon cancer: a systematic review. Can J Surg 2007; 50(1):48-57.
33. Kerkhoffs GM, Handoll HH, de Bie R, Rowe BH, Struijs PA. Surgical versus conservative treatment for acute injuries of the lateral ligament complex of the ankle in adults. Cochrane Database Syst Rev 2007(2):CD000380.
34. Kunadian B, Dunning J, Vijayalakshmi K, Thornley AR, de Belder MA. Meta-analysis of randomized trials comparing anti-embolic devices with standard PCI for improving myocardial reperfusion in patients with acute myocardial infarction. Catheter Cardiovasc Interv 2007; 69(4):488-96.
35. Lemos HP, Jr., Atallah AN. Does the use of paclitaxel or rapamycin-eluting stent decrease further need for coronary-artery bypass grafting when compared with bare-metal stent? Sao Paulo Med J 2007; 125(4):242-5.
36. Lens MB, Nathan P, Bataille V. Excision margins for primary cutaneous melanoma: updated pooled analysis of randomized controlled trials. Arch Surg 2007; 142(9):885-91; discussion 91-3.
37. Liang Y, Li G, Chen P, Yu J. Laparoscopic versus open colorectal resection for cancer: a meta-analysis of results of randomized controlled trials on recurrence. Eur J Surg Oncol 2007; 34(11):1217-24.
38. Luebke T, Aleksic M, Brunkwall J. Meta-analysis of randomized trials comparing carotid endarterectomy and endovascular treatment. Eur J Vasc Endovasc Surg 2007; 34(4):470-9.
39. Michalski CW, Kleeff J, Wente MN, Diener MK, Buchler MW, Friess H. Systematic review and meta-analysis of standard and extended lymphadenectomy in pancreaticoduodenectomy for pancreatic cancer. Br J Surg 2007; 94(3):265-73.
40. Mirza SK, Deyo RA. Systematic review of randomized trials comparing lumbar fusion surgery to nonoperative care for treatment of chronic back pain. Spine (Phila Pa 1976) 2007; 32(7):816-23.
41. Morrison J, Swanton A, Collins S, Kehoe S. Chemotherapy versus surgery for initial treatment in advanced ovarian epithelial cancer. Cochrane Database Syst Rev 2007(4):CD005343.
42. Moss AC, Morris E, Leyden J, MacMathuna P. Do the benefits of metal stents justify the costs? A systematic review and meta-analysis of trials comparing endoscopic stents for malignant biliary obstruction. Eur J Gastroenterol Hepatol 2007; 19(12):1125-39.
43. Moss AC, Morris E, Leyden J, MacMathuna P. Malignant distal biliary obstruction: a systematic review and meta-analysis of endoscopic and surgical bypass results. Cancer Treat Rev 2007; 33(2):213-21.
44. Mwipatayi BP, Hockings A, Hofmann M, Garbowski M, Sieunarine K. Balloon angioplasty compared with stenting for treatment of femoropopliteal occlusive disease: a meta-analysis. J Vasc Surg 2007; 47(2):461-9.
45. Nabi G, Cook J, N'Dow J, McClinton S. Outcomes of stenting after uncomplicated ureteroscopy: systematic review and meta-analysis. BMJ 2007; 334(7593):572.
46. Nabi G, Downey P, Keeley F, Watson G, McClinton S. Extra-corporeal shock wave lithotripsy (ESWL) versus ureteroscopic management for ureteric calculi. Cochrane Database Syst Rev 2007(1):CD006029.
47. Novara G, Ficarra V, Boscolo-Berto R, Secco S, Cavalleri S, Artibani W. Tension-free midurethral slings in the treatment of female stress urinary incontinence: a systematic review and meta-analysis of randomized controlled trials of effectiveness. Eur Urol 2007; 52(3):663-78.
48. Pham C, Greenwood J, Cleland H, Woodruff P, Maddern G. Bioengineered skin substitutes for the management of burns: a systematic review. Burns 2007; 33(8):946-57.
49. Purkayastha S, Tilney HS, Georgiou P, Athanasiou T, Tekkis PP, Darzi AW. Laparoscopic cholecystectomy versus mini-laparotomy cholecystectomy: a meta-analysis of randomised control trials. Surg Endosc 2007; 21(8):1294-300.
50. Restrepo C, Parvizi J, Dietrich T, Einhorn TA. Safety of simultaneous bilateral total knee arthroplasty. A meta-analysis. J Bone Joint Surg Am 2007; 89(6):1220-6.
51. Sajid MS, Hunte S, Hippolyte S, Kiri VA, Maringe C, Baig MK. Comparison of surgical vs chemical sphincterotomy using botulinum toxin for the treatment of chronic anal fissure: a meta-analysis. Int J Gynaecol Obstet 2007; 99(Suppl 1):S40-6.
52. Scholten RJ, Mink van der Molen A, Uitdehaag BM, Bouter LM, de Vet HC. Surgical treatment options for carpal tunnel syndrome. Cochrane Database Syst Rev 2007(4):CD003905.
53. Svilaas T, van der Horst IC, Zijlstra F. A quantitative estimate of bare-metal stenting compared with balloon angioplasty in patients with acute myocardial infarction: angiographic measures in relation to clinical outcome. Heart 2007; 93(7):792-800.
54. Tan A, Liao C, Mo Z, Cao Y. Meta-analysis of holmium laser enucleation versus transurethral resection of the prostate for symptomatic prostatic obstruction. Br J Surg 2007; 94(10):1201-8.
55. Tan EK, Cornish J, Darzi AW, Papagrigoriadis S, Tekkis PP. Meta-analysis of short-term outcomes of randomized controlled trials of LigaSure vs conventional hemorrhoidectomy. Arch Surg 2007; 142(12):1209-18
56. Tjandra JJ, Chan MK. Systematic review on the procedure for prolapse and hemorrhoids (stapled hemorrhoidopexy). Dis Colon Rectum 2007; 50(6):878-92.
57. Tripathi D, Graham C, Hayes PC. Variceal band ligation versus beta-blockers for primary prevention of variceal bleeding: a meta-analysis. Eur J Gastroenterol Hepatol 2007; 19(10):835-45.
58. Verma D, Kapadia A, Adler DG. Pure versus mixed electrosurgical current for endoscopic biliary sphincterotomy: a meta-analysis of adverse outcomes. Gastrointest Endosc 2007; 66(2):283-90.
59. Wakai A, O'Sullivan RG, McCabe G. Simple aspiration versus intercostal tube drainage for primary spontaneous pneumothorax in adults. Cochrane Database Syst Rev 2007(1):CD004479.
60. Wijeysundera HC, Vijayaraghavan R, Nallamothu BK, Foody JM, Krumholz HM, Phillips CO, et al. Rescue angioplasty or repeat fibrinolysis after failed fibrinolytic therapy for ST-segment myocardial infarction: a meta-analysis of randomized trials. J Am Coll Cardiol 2007; 49(4):422-30.
61. Zlowodzki M, Chan S, Bhandari M, Kalliainen L, Schubert W. Anterior transposition compared with simple decompression for treatment of cubital tunnel syndrome. A meta-analysis of randomized, controlled trials. J Bone Joint Surg Am 2007; 89(12):2591-8.
62. Abraha I, Binda GA, Montedori A, Arezzo A, Cirocchi R. Laparoscopic versus open resection for sigmoid diverticulitis. Cochrane Database Syst Rev 2017; 11(doi):CD009277.
63. Agarwal N, Jain A, Garg J, Mojadidi MK, Mahmoud AN, Patel NK, et al. Staged versus index procedure complete revascularization in ST-elevation myocardial infarction: A meta-analysis. J Interv Cardiol 2017; 30(5):397-404.
64. Al Otaibi A, Gupta S, Belley-Cote EP, Alsagheir A, Spence J, Parry D, et al. Mini-thoracotomy vs. conventional sternotomy mitral valve surgery: a systematic review and meta-analysis. J Cardiovasc Surg (Torino) 2017; 58(3):489-96.
65. Alcelik I, Blomfield M, Ozturk C, Soni A, Charity R, Acornley A. A comparison of short term radiological alignment outcomes of the patient specific and standard instrumentation for primary total knee arthroplasty: A systematic review and meta-analysis. Acta Orthop Traumatol Turc 2017; 51(3):215-22.
66. Allievi N, Ceresoli M, Fugazzola P, Montori G, Coccolini F, Ansaloni L. Endoscopic Stenting as Bridge to Surgery versus Emergency Resection for Left-Sided Malignant Colorectal Obstruction: An Updated Meta-Analysis. Int J Surg Oncol 2017; 2017(19-20):2863272.
67. Antoniou GA, Georgiadis GS, Antoniou SA, Makar RR, Smout JD, Torella F. Bypass surgery for chronic lower limb ischaemia. Cochrane Database Syst Rev 2017; 4(3):CD002000.
68. Antoniou SA, Garcia-Alamino JM, Hajibandeh S, Hajibandeh S, Weitzendorfer M, Muysoms FE, et al. Single-incision surgery trocar-site hernia: an updated systematic review meta-analysis with trial sequential analysis by the Minimally Invasive Surgery Synthesis of Interventions Outcomes Network (MISSION). Surg Endosc 2018; 32(1):14-23.
69. Arezzo A, Passera R, Lo Secco G, Verra M, Bonino MA, Targarona E, et al. Stent as bridge to surgery for left-sided malignant colonic obstruction reduces adverse events and stoma rate compared with emergency surgery: results of a systematic review and meta-analysis of randomized controlled trials. Gastrointest Endosc 2017; 86(3):416-26.
70. Bavishi C, Baber U, Panwar S, Pirrotta S, Dangas GD, Moreno P, et al. Efficacy and safety of everolimus and zotarolimus-eluting stents versus first-generation drug-eluting stents in patients with diabetes: A meta-analysis of randomized trials. Int J Cardiol 2017; 230(2):310-18.
71. Bravo CA, Hirji SA, Bhatt DL, Kataria R, Faxon DP, Ohman EM, et al. Complete versus culprit-only revascularisation in ST elevation myocardial infarction with multi-vessel disease. Cochrane Database Syst Rev 2017; 5(Suppl 2):CD011986.
72. Bundhun PK, Yanamala CM, Huang WQ. Comparing Stent Thrombosis associated with Zotarolimus Eluting Stents versus Everolimus Eluting Stents at 1 year follow up: a systematic review and meta-analysis of 6 randomized controlled trials. BMC Cardiovasc Disord 2017; 17(1):84.
73. Burrage M, Moore P, Cole C, Cox S, Lo WC, Rafter A, et al. Transcatheter Aortic Valve Replacement is Associated with Comparable Clinical Outcomes to Open Aortic Valve Surgery but with a Reduced Length of In-Patient Hospital Stay: A Systematic Review and Meta-Analysis of Randomised Trials. Heart Lung Circ 2017; 26(3):285-95.
74. Carson-Chahhoud KV, Wakai A, van Agteren JE, Smith BJ, McCabe G, Brinn MP, et al. Simple aspiration versus intercostal tube drainage for primary spontaneous pneumothorax in adults. Cochrane Database Syst Rev 2017; 9(1):CD004479.
75. Chapman SJ, Wood B, Drake TM, Young N, Jayne DG. Systematic review and meta-analysis of prophylactic mesh during primary stoma formation to prevent parastomal hernia. Dis Colon Rectum 2017; 60(1):107-15.
76. Charoenkwan K, Iheozor-Ejiofor Z, Rerkasem K, Matovinovic E. Scalpel versus electrosurgery for major abdominal incisions. Cochrane Database Syst Rev 2017; 6(1):CD005987.
77. Charoenkwan K, Kietpeerakool C. Retroperitoneal drainage versus no drainage after pelvic lymphadenectomy for the prevention of lymphocyst formation in women with gynaecological malignancies. Cochrane Database Syst Rev 2017; 6(6):CD007387.
78. Chen BL, Guo JB, Zhang HW, Zhang YJ, Zhu Y, Zhang J, et al. Surgical versus non-operative treatment for lumbar disc herniation: a systematic review and meta-analysis. Clin Rehabil 2018; 32(2):146-60.
79. Cheng JW, Li Y, Xing WQ, Lv HW, Wang HR. Laparoscopic Heller myotomy is not superior to pneumatic dilation in the management of primary achalasia: Conclusions of a systematic review and meta-analysis of randomized controlled trials. Medicine (Baltimore) 2017; 96(7):e6114.
80. Cheng Y, Briarava M, Lai M, Wang X, Tu B, Cheng N, et al. Pancreaticojejunostomy versus pancreaticogastrostomy reconstruction for the prevention of postoperative pancreatic fistula following pancreaticoduodenectomy. Cochrane Database Syst Rev 2017; 9(1):CD012257.
81. Cheng Y, Xiong X, Lu J, Wu S, Zhou R, Cheng N. Early versus delayed appendicectomy for appendiceal phlegmon or abscess. Cochrane Database Syst Rev 2017; 6(26):CD011670.
82. Ciccotti MC, Secrist E, Tjoumakaris F, Ciccotti MG, Freedman KB. Anatomic Anterior Cruciate Ligament Reconstruction via Independent Tunnel Drilling: A Systematic Review of Randomized Controlled Trials Comparing Patellar Tendon and Hamstring Autografts. Arthroscopy 2017; 33(5):1062-71 e5.
83. Cirocchi R, Di Saverio S, Weber DG, Tabola R, Abraha I, Randolph J, et al. Laparoscopic lavage versus surgical resection for acute diverticulitis with generalised peritonitis: a systematic review and meta-analysis. Tech Coloproctol 2017; 21(2):93-110.
84. Cohen R, Le Roux CW, Junqueira S, Ribeiro RA, Luque A. Roux-En-Y Gastric Bypass in Type 2 Diabetes Patients with Mild Obesity: a Systematic Review and Meta-analysis. Obes Surg 2017; 27(10):2733-39.
85. Collet C, Asano T, Miyazaki Y, Tenekecioglu E, Katagiri Y, Sotomi Y, et al. Late thrombotic events after bioresorbable scaffold implantation: a systematic review and meta-analysis of randomized clinical trials. Eur Heart J 2017; 38(33):2559-66.
86. Coppolino G, Pisano A, Rivoli L, Bolignano D. Renal denervation for resistant hypertension. Cochrane Database Syst Rev 2017; 2(1):CD011499.
87. Cornille JB, Pathak S, Daniels IR, Smart NJ. Prophylactic mesh use during primary stoma formation to prevent parastomal hernia. Ann R Coll Surg Engl 2017; 99(1):2-11.
88. Cross AJ, Buchwald PL, Frizelle FA, Eglinton TW. Meta-analysis of prophylactic mesh to prevent parastomal hernia. Br J Surg 2017; 104(3):179-86.
89. Cui K, Lyu S, Song X, Yuan F, Xu F, Zhang M, et al. Drug-eluting balloon versus bare-mental stent and drug-eluting stent for de novo coronary artery disease: A systematic review and meta-analysis of 14 randomized controlled trials. PLoS One 2017; 12(4):e0176365.
90. Dahal K, Rijal J, Shahukhal R, Sharma S, Watti H, Azrin M, et al. Comparison of manual compression and vascular hemostasis devices after coronary angiography or percutaneous coronary intervention through femoral artery access: A meta-analysis of randomized controlled trials. Cardiovasc Revasc Med 2018; 19(2):151-62.
91. Dai L, Shuai J. Laparoscopic versus open appendectomy in adults and children: A meta-analysis of randomized controlled trials. United European Gastroenterol J 2017; 5(4):542-53.
92. De Rosa S, Polimeni A, Sabatino J, Indolfi C. Long-term outcomes of coronary artery bypass grafting versus stent-PCI for unprotected left main disease: a meta-analysis. BMC Cardiovasc Disord 2017; 17(1):240.
93. Deng L, Xiong J, Xia Q. Single-incision versus conventional three-incision laparoscopic appendectomy: A meta-analysis of randomized controlled trials. J Evid Based Med 2017; 10(3):196-206.
94. Deng S, Sun Z, Zhang C, Chen G, Li J. Surgical Treatment Versus Conservative Management for Acute Achilles Tendon Rupture: A Systematic Review and Meta-Analysis of Randomized Controlled Trials. J Foot Ankle Surg 2017; 56(6):1236-43.
95. Diniz JM, Botelho RV. Is fusion necessary for thoracolumbar burst fracture treated with spinal fixation? A systematic review and meta-analysis. J Neurosurg Spine 2017; 27(5):584-92.
96. Dong L, Xu Z, Chen X, Wang D, Li D, Liu T, et al. The change of adjacent segment after cervical disc arthroplasty compared with anterior cervical discectomy and fusion: a meta-analysis of randomized controlled trials. Spine J 2017; 17(10):1549-58.
97. Dressler J, Jorgensen LN. The use of expanding ports in laparo-endoscopic single-site surgery may cause more pain: a meta-analysis of randomized clinical trials. Surg Endosc 2017; 31(11):4400-11.
98. Du X, Wu JM, Hu ZW, Wang F, Wang ZG, Zhang C, et al. Laparoscopic Nissen (total) versus anterior 180 degrees fundoplication for gastro-esophageal reflux disease: A meta-analysis and systematic review. Medicine (Baltimore) 2017; 96(37):e8085.
99. El-Hayek G, Bangalore S, Casso Dominguez A, Devireddy C, Jaber W, Kumar G, et al. Meta-Analysis of Randomized Clinical Trials Comparing Biodegradable Polymer Drug-Eluting Stent to Second-Generation Durable Polymer Drug-Eluting Stents. JACC Cardiovasc Interv 2017; 10(5):462-73.
100. Emile SH, Elfeki H. Desarda's technique versus Lichtenstein technique for the treatment of primary inguinal hernia: a systematic review and meta-analysis of randomized controlled trials. Hernia 2018; 22(3):385-95.
101. Farag S, Rehman S, Sains P, Baig MK, Sajid MS. Early vs delayed closure of loop defunctioning ileostomy in patients undergoing distal colorectal resections: an integrated systematic review and meta-analysis of published randomized controlled trials. Colorectal Dis 2017; 19(12):1050-57.
102. Frost JA, Webster KE, Bryant A, Morrison J. Lymphadenectomy for the management of endometrial cancer. Cochrane Database Syst Rev 2017; 10(8):CD007585.
103. Gaffar R, Habib B, Filion KB, Reynier P, Eisenberg MJ. Optimal Timing of Complete Revascularization in Acute Coronary Syndrome: A Systematic Review and Meta-Analysis. J Am Heart Assoc 2017; 6(4):10
104. Gao K, Sun Y, Yang M, Han L, Chen L, Hu W, et al. Efficacy and safety of polymer-free stent versus polymer-permanent drug-eluting stent in patients with acute coronary syndrome: a meta-analysis of randomized control trials. BMC Cardiovasc Disord 2017; 17(1):194.
105. Gao L, Liu Y, Sun Z, Wang Y, Cao F, Chen Y. Percutaneous coronary intervention using drug-eluting stents versus coronary artery bypass graft surgery in left main coronary artery disease an updated meta-analysis of randomized clinical trials. Oncotarget 2017; 8(39):66449-57.
106. Gao YC, Chen J, Qin Q, Chen H, Wang W, Zhao J, et al. Efficacy and safety of laparoscopic bile duct exploration versus endoscopic sphincterotomy for concomitant gallstones and common bile duct stones: A meta-analysis of randomized controlled trials. Medicine (Baltimore) 2017; 96(37):e7925.
107. Garg A, Rao SV, Agrawal S, Theodoropoulos K, Mennuni M, Sharma A, et al. Meta-Analysis of Randomized Controlled Trials of Percutaneous Coronary Intervention With Drug-Eluting Stents Versus Coronary Artery Bypass Grafting in Left Main Coronary Artery Disease. Am J Cardiol 2017; 119(12):1942-48.
108. Giacoppo D, Colleran R, Cassese S, Frangieh AH, Wiebe J, Joner M, et al. Percutaneous Coronary Intervention vs Coronary Artery Bypass Grafting in Patients With Left Main Coronary Artery Stenosis: A Systematic Review and Meta-analysis. JAMA Cardiol 2017; 2(10):1079-88.
109. Gimzewska M, Jackson AI, Yeoh SE, Clarke M. Totally percutaneous versus surgical cut-down femoral artery access for elective bifurcated abdominal endovascular aneurysm repair. Cochrane Database Syst Rev 2017; 2(2):CD010185.
110. Glazener CM, Cooper K, Mashayekhi A. Anterior vaginal repair for urinary incontinence in women. Cochrane Database Syst Rev 2017; 7(6):CD001755.
111. Gozdek M, Pawliszak W, Hagner W, Zalewski P, Kowalewski J, Paparella D, et al. Systematic review and meta-analysis of randomized controlled trials assessing safety and efficacy of posterior pericardial drainage in patients undergoing heart surgery. J Thorac Cardiovasc Surg 2017; 153(4):865-75 e12.
112. Grant MC, Yang D, Wu CL, Makary MA, Wick EC. Impact of enhanced recovery after surgery and fast track surgery pathways on healthcare-associated infections: results from a systematic review and meta-analysis. Ann Surg 2017; 265(1):68-79.
113. Hao XY, Shen YF, Wei YG, Liu F, Li HY, Li B. Safety and effectiveness of day-surgery laparoscopic cholecystectomy is still uncertain: meta-analysis of eight randomized controlled trials based on GRADE approach. Surgical Endoscopy and Other Interventional Techniques 2017; 31(12):4950-63.
114. Haueter R, Schutz T, Raptis DA, Clavien PA, Zuber M. Meta-analysis of single-port versus conventional laparoscopic cholecystectomy comparing body image and cosmesis. Br J Surg 2017; 104(9):1141-59.
115. Huo ZC, Liu G, Li XY, Liu F, Fan WJ, Guan RH, et al. Use of a disposable circumcision suture device versus conventional circumcision: a systematic review and meta-analysis. Asian Journal of Andrology 2017; 19(3):362-67.
116. Huttner FJ, Probst P, Knebel P, Strobel O, Hackert T, Ulrich A, et al. Meta-analysis of prophylactic abdominal drainage in pancreatic surgery. Br J Surg 2017; 104(6):660-68.
117. Ilic D, Evans SM, Allan CA, Jung JH, Murphy D, Frydenberg M. Laparoscopic and robotic-assisted versus open radical prostatectomy for the treatment of localised prostate cancer. Cochrane Database Syst Rev 2017; 9(5):CD009625.
118. Jin B, Chen MT, Fei YT, Du SD, Mao YL. Safety and efficacy for laparoscopic versus open hepatectomy: A meta-analysis. Surg Oncol 2018; 27(2):A26-A34.
119. Kakkos SK, Kakisis I, Tsolakis IA, Geroulakos G. Endarterectomy achieves lower stroke and death rates compared with stenting in patients with asymptomatic carotid stenosis. J Vasc Surg 2017; 66(2):607-17.
120. Kallidonis P, Ntasiotis P, Knoll T, Sarica K, Papatsoris A, Somani BK, et al. Minimally Invasive Surgical Ureterolithotomy Versus Ureteroscopic Lithotripsy for Large Ureteric Stones: A Systematic Review and Meta-analysis of the Literature. Eur Urol Focus 2017; 3(6):554-66.
121. Khan AR, Golwala H, Tripathi A, Riaz H, Kumar A, Flaherty MP, et al. Meta-analysis of Percutaneous Coronary Intervention Versus Coronary Artery Bypass Grafting in Left Main Coronary Artery Disease. Am J Cardiol 2017; 119(12):1949-56.
122. Khan SU, Rahman H, Arshad A, Khan MU, Lekkala M, Yang T, et al. Percutaneous Coronary Intervention Versus Surgery in Left Main Stenosis-A Meta-Analysis and Systematic Review of Randomised Controlled Trials. Heart Lung Circ 2018; 27(2):138-46.
123. Kim JS, Kwon SH, Lee EJ, Yoon YJ. Can Intracapsular Tonsillectomy Be an Alternative to Classical Tonsillectomy? A Meta-analysis. J Int Med Res 2017; 45(3):897-903. doi: 10.1177/0300060517701356. Epub 2017 Apr 18.
124. Kirmani BH, Jones SG, Malaisrie SC, Chung DA, Williams RJ. Limited versus full sternotomy for aortic valve replacement. Cochrane Database Syst Rev 2017; 4(2):CD011793.
125. Klugarova J, Hood V, Bath-Hextall F, Klugar M, Mareckova J, Kelnarova Z. Effectiveness of surgery for adults with hallux valgus deformity: a systematic review. JBI Database System Rev Implement Rep 2017; 15(6):1671-710.
126. Komaei I, Navarra G, Curro G. Three-Dimensional Versus Two-Dimensional Laparoscopic Cholecystectomy: A Systematic Review. J Laparoendosc Adv Surg Tech A 2017; 27(8):790-94.
127. Kunath F, Schmidt S, Krabbe LM, Miernik A, Dahm P, Cleves A, et al. Partial nephrectomy versus radical nephrectomy for clinical localised renal masses. Cochrane Database Syst Rev 2017; 5(1):CD012045.
128. Lauridsen SV, Tonnesen H, Jensen BT, Neuner B, Thind P, Thomsen T. Complications and health-related quality of life after robot-assisted versus open radical cystectomy: a systematic review and meta-analysis of four RCTs. Syst Rev 2017; 6(1):150.
129. Li AB, Zhang WJ, Wang J, Guo WJ, Wang XH, Zhao YM. Intramedullary and extramedullary fixations for the treatment of unstable femoral intertrochanteric fractures: a meta-analysis of prospective randomized controlled trials. International Orthopaedics 2017; 41(2):403-13.
130. Li C, Dai Z, Gong Y, Xie B, Wang B. A systematic review and meta-analysis of randomized controlled trials comparing hysteroscopic morcellation with resectoscopy for patients with endometrial lesions. Int J Gynaecol Obstet 2017; 136(1):6-12.
131. Li Y, Yang JJ, Zhu SH, Xu B, Wang L. Long-term efficacy and safety of carotid artery stenting versus endarterectomy: A meta-analysis of randomized controlled trials. PLoS One 2017; 12(7):e0180804.
132. Liu L, Su SW, Sun HY. Safety of Extracranial-Intracranial Arterial Bypass in the Treatment of Moyamoya Disease. J Invest Surg 2017; 31(1):14-23.
133. Locke JA, Noparast M, Afshar K. Treatment of varicocele in children and adolescents: A systematic review and meta-analysis of randomized controlled trials. J Pediatr Urol 2017; 13(5):437-45.
134. Lopez-Cano M, Brandsma HT, Bury K, Hansson B, Kyle-Leinhase I, Alamino JG, et al. Prophylactic mesh to prevent parastomal hernia after end colostomy: a meta-analysis and trial sequential analysis. Hernia 2017; 21(2):177-89.
135. Lu W, Zhu Y, Han Z, Wang X, Wang X, Qiu C. Drug-coated balloon in combination with bare metal stent strategy for de novo coronary artery disease: A PRISMA-compliant meta-analysis of randomized clinical trials. Medicine (Baltimore) 2017; 96(12):e6397.
136. Ma XL, Zhao XW, Ma JX, Li F, Wang Y, Lu B. Effectiveness of surgery versus conservative treatment for lumbar spinal stenosis: A system review and meta-analysis of randomized controlled trials. Int J Surg 2017; 44(7):329-38.
137. Mahmoud AN, Barakat AF, Elgendy AY, Schneibel E, Mentias A, Abuzaid A, et al. Long-Term Efficacy and Safety of Everolimus-Eluting Bioresorbable Vascular Scaffolds Versus Everolimus-Eluting Metallic Stents: A Meta-Analysis of Randomized Trials. Biomed Inform Insights 2017; 9:1178222617697975.
138. Maher C, Baessler K, Glazener CM, Adams EJ, Hagen S. Surgical management of pelvic organ prolapse in women. Cochrane Database Syst Rev 2007(3):CD004014.
139. Mannu GS, Sudul MK, Bettencourt-Silva JH, Cumber E, Li F, Clark AB, et al. Closure methods of the appendix stump for complications during laparoscopic appendectomy. Cochrane Database Syst Rev 2017; 11(3):CD006437.
140. McClure GR, Belley-Cote EP, Jaffer IH, Dvirnik N, An KR, Fortin G, et al. Surgical ablation of atrial fibrillation: a systematic review and meta-analysis of randomized controlled trials. Biomolecules 2017; 7(4).
141. Menahem B, Vallois A, Alves A, Lubrano J. Prophylactic pelvic drainage after rectal resection with extraperitoneal anastomosis: is it worthwhile? A meta-analysis of randomized controlled trials. Int J Colorectal Dis 2017; 32(11):1531-38.
142. Molegraaf M, Kaufmann R, Lange J. Comparison of self-gripping mesh and sutured mesh in open inguinal hernia repair: A meta-analysis of long-term results. Surgery 2017; 163(2):351-60.
143. Montone RA, Niccoli G, De Marco F, Minelli S, D'Ascenzo F, Testa L, et al. Temporal Trends in Adverse Events After Everolimus-Eluting Bioresorbable Vascular Scaffold Versus Everolimus-Eluting Metallic Stent Implantation: A Meta-Analysis of Randomized Controlled Trials. Circulation 2017; 135(22):2145-54.
144. Moore P, Burrage M, Garrahy P, Lim R, McCann A, Camuglia A. Drug-Eluting Stents Versus Coronary Artery Bypass Grafts for Left Main Coronary Disease: A Meta-Analysis and Review of Randomised Controlled Trials. Heart Lung Circ 2017; 27(12):1437-45.
145. Moresoli P, Habib B, Reynier P, Secrest MH, Eisenberg MJ, Filion KB. Carotid Stenting Versus Endarterectomy for Asymptomatic Carotid Artery Stenosis: A Systematic Review and Meta-Analysis. Stroke 2017; 48(8):2150-57.
146. Nairooz R, Saad M, Elgendy IY, Mahmoud AN, Habash F, Sardar P, et al. Long-term outcomes of provisional stenting compared with a two-stent strategy for bifurcation lesions: a meta-analysis of randomised trials. Heart 2017; 103(18):1427-34.
147. Osland E, Yunus RM, Khan S, Memon B, Memon MA. Weight Loss Outcomes in Laparoscopic Vertical Sleeve Gastrectomy (LVSG) Versus Laparoscopic Roux-en-Y Gastric Bypass (LRYGB) Procedures: A Meta-Analysis and Systematic Review of Randomized Controlled Trials. Surg Laparosc Endosc Percutan Tech 2017; 27(1):8-18.
148. Osland E, Yunus RM, Khan S, Memon B, Memon MA. Changes in Non-Diabetic Comorbid Disease Status Following Laparoscopic Vertical Sleeve Gastrectomy (LVSG) Versus Laparoscopic Roux-En-Y Gastric Bypass (LRYGB) Procedures: a Systematic Review of Randomized Controlled Trials. Obes Surg 2017; 27(5):1208-21.
149. Osland E, Yunus RM, Khan S, Memon B, Memon MA. Diabetes improvement and resolution following laparoscopic vertical sleeve gastrectomy (LVSG) versus laparoscopic Roux-en-Y gastric bypass (LRYGB) procedures: a systematic review of randomized controlled trials. Surg Endosc 2017; 31(4):1952-63.
150. Palmerini T, Serruys P, Kappetein AP, Genereux P, Riva DD, Reggiani LB, et al. Clinical outcomes with percutaneous coronary revascularization vs coronary artery bypass grafting surgery in patients with unprotected left main coronary artery disease: A meta-analysis of 6 randomized trials and 4,686 patients. Am Heart J 2017; 190(doi):54-63.
151. Patel SV, Zhang L, Chadi SA, Wexner SD. Prophylactic mesh to prevent parastomal hernia: a meta-analysis of randomized controlled studies. Tech Coloproctol 2017; 21(1):5-13.
152. Patterson T, Currie P, Patterson S, Patterson P, Meek C, McMaster R. A systematic review and meta-analysis of the post-operative adverse effects associated with mosquito net mesh in comparison to commercial hernia mesh for inguinal hernia repair in low income countries. Hernia 2017; 21(3):397-405.
153. Pedziwiatr M, Malczak P, Mizera M, Witowski J, Torbicz G, Major P, et al. There is no difference in outcome between laparoscopic and open surgery for rectal cancer: a systematic review and meta-analysis on short- and long-term oncologic outcomes. Techniques in Coloproctology 2017; 21(8):595-604.
154. Polimeni A, Anadol R, Munzel T, Indolfi C, De Rosa S, Gori T. Long-term outcome of bioresorbable vascular scaffolds for the treatment of coronary artery disease: a meta-analysis of RCTs. BMC Cardiovasc Disord 2017;17(1):147.
155. Putzu A, Gallo M, Martino EA, Ferrari E, Pedrazzini G, Moccetti T, et al. Coronary artery bypass graft surgery versus percutaneous coronary intervention with drug-eluting stents for left main coronary artery disease: A meta-analysis of randomized trials. Int J Cardiol 2017; 241(1):142-48.
156. Pynnonen M, Brinkmeier JV, Thorne MC, Chong LY, Burton MJ. Coblation versus other surgical techniques for tonsillectomy. Cochrane Database Syst Rev 2017; 8:CD004619.
157. Qian C, Feng H, Cao J, Wei B, Wang Y. Meta-Analysis of Randomized Control Trials Comparing Drug-Eluting Stents Versus Coronary Artery Bypass Grafting for Significant Left Main Coronary Narrowing. Am J Cardiol 2017; 119(9):1338-43.
158. Redden MD, Chin TY, van Driel ML. Surgical versus non-surgical management for pleural empyema. Cochrane Database Syst Rev 2017; 3(6):CD010651.
159. Rezende FC, Moraes VY, Franciozi CE, Debieux P, Luzo MV, Belloti JC. One-incision versus two-incision techniques for arthroscopically assisted anterior cruciate ligament reconstruction in adults. Cochrane Database Syst Rev 2017; 12(10):CD010875.
160. Ricci C, Casadei R, Taffurelli G, Pacilio CA, Beltrami D, Minni F. Is pancreaticogastrostomy safer than pancreaticojejunostomy after pancreaticoduodenectomy? A meta-regression analysis of randomized clinical trials. Pancreatology 2017; 17(5):805-13.
161. Ryosa A, Laimi K, Aarimaa V, Lehtimaki K, Kukkonen J, Saltychev M. Surgery or conservative treatment for rotator cuff tear: a meta-analysis. Disabil Rehabil 2017; 39(14):1357-63.
162. Saber AA, Shoar S, Almadani MW, Zundel N, Alkuwari MJ, Bashah MM, et al. Efficacy of First-Time Intragastric Balloon in Weight Loss: a Systematic Review and Meta-analysis of Randomized Controlled Trials. Obes Surg 2017; 27(2):277-87.
163. Sajid MS, Rathore MA, Sains P, Singh KK. A systematic review of clinical effectiveness of wound edge protector devices in reducing surgical site infections in patients undergoing abdominal surgery. Updates Surg 2017; 69(1):21-28.
164. Sakran JV, Mylonas KS, Gryparis A, Stawicki SP, Burns CJ, Matar MM, et al. Operation versus antibiotics-The "appendicitis conundrum" continues: A meta-analysis. Journal of Trauma and Acute Care Surgery 2017; 82(6):1129-37.
165. Sardar P, Chatterjee S, Aronow HD, Kundu A, Ramchand P, Mukherjee D, et al. Carotid Artery Stenting Versus Endarterectomy for Stroke Prevention: A Meta-Analysis of Clinical Trials. J Am Coll Cardiol 2017; 69(18):2266-75.
166. Sardar P, Giri J, Elmariah S, Chatterjee S, Kolte D, Kundu A, et al. Meta-Analysis of Drug-Eluting Stents Versus Coronary Artery Bypass Grafting in Unprotected Left Main Coronary Narrowing. Am J Cardiol 2017; 119(11):1746-52.
167. Sarode D, Bari DA, Cain AC, Syed MI, Williams AT. The benefit of silicone stents in primary endonasal dacryocystorhinostomy: a systematic review and meta-analysis. Clin Otolaryngol 2017; 42(2):307-14.
168. Scheuermann U, Niebisch S, Lyros O, Jansen-Winkeln B, Gockel I. Transabdominal Preperitoneal (TAPP) versus Lichtenstein operation for primary inguinal hernia repair - A systematic review and meta-analysis of randomized controlled trials. BMC Surg 2017; 17(1):55.
169. Schuurmans J, Goslings JC, Schepers T. Operative management versus non-operative management of rib fractures in flail chest injuries: a systematic review. Eur J Trauma Emerg Surg 2017; 43(2):163-68.
170. Shaikh FM, Stewart PM, Walsh SR, Davies RJ. Laparoscopic peritoneal lavage or surgical resection for acute perforated sigmoid diverticulitis: A systematic review and meta-analysis. Int J Surg 2017; 38(2):130-37.
171. Shangguan L, Ning GZ, Tang Y, Wang Z, Luo ZJ, Zhou Y. Discover cervical disc arthroplasty versus anterior cervical discectomy and fusion in symptomatic cervical disc diseases: A meta-analysis. PLoS One 2017; 12(3):e0174822.
172. Sharma SP, Dahal K, Khatra J, Rosenfeld A, Lee J. Percutaneous coronary intervention vs coronary artery bypass grafting for left main coronary artery disease? A systematic review and meta-analysis of randomized controlled trials. Cardiovasc Ther 2017; 35(3):820-27.
173. Sharma SP, Sangha RS, Dahal K, Krishnamoorthy P. The role of empiric superior vena cava isolation in atrial fibrillation: a systematic review and meta-analysis of randomized controlled trials. J Interv Card Electrophysiol 2017; 48(1):61-67.
174. Sun P, Cheng X, Deng S, Hu Q, Sun Y, Zheng Q. Mesh fixation with glue versus suture for chronic pain and recurrence in Lichtenstein inguinal hernioplasty. Cochrane Database Syst Rev 2017; 2(3):CD010814.
175. Tan C, Ocampo O, Ong R, Tan KS. Comparison of one stage laparoscopic cholecystectomy combined with intra-operative endoscopic sphincterotomy versus two-stage pre-operative endoscopic sphincterotomy followed by laparoscopic cholecystectomy for the management of pre-operatively diagnosed patients with common bile duct stones: a meta-analysis. Surg Endosc 2017; 32(2):770-78.
176. Tan CC, Wang HF, Ji JL, Tan MS, Tan L, Yu JT. Endovascular treatment versus intravenous thrombolysis for acute ischemic stroke: a quantitative review and meta-analysis of 21 randomized trials. Mol Neurobiol 2017; 54(2):1369-78.
177. Tang Q, Shang P, Zheng G, Xu HZ, Liu HX. Extramedullary versus intramedullary femoral alignment technique in total knee arthroplasty: a meta-analysis of randomized controlled trials. J Orthop Surg Res 2017; 12(1):82.
178. Tong MJ, Tang Q, Wang CG, Xiang GH, Chen Q, Xu HZ, et al. Efficacy of Using Intermediate Screws in Short-Segment Fixation for Thoracolumbar Fractures: A Meta-Analysis of Randomized Controlled Trials. Transl Lung Cancer Res 2017; 6(5):588-99.
179. Tse F, Yuan Y, Moayyedi P, Leontiadis GI, Barkun AN. Double-guidewire technique in difficult biliary cannulation for the prevention of post-ERCP pancreatitis: a systematic review and meta-analysis. Endoscopy 2017; 49(1):15-26.
180. Upadhaya S, Baniya R, Madala S, Subedi SK, Khan J, Velagapudi RK, et al. Drug-eluting stent placement versus coronary artery bypass surgery for unprotected left main coronary artery disease: A meta-analysis of randomized controlled trials. J Card Surg 2017; 32(2):70-79.
181. van Agteren JE, Hnin K, Grosser D, Carson KV, Smith BJ. Bronchoscopic lung volume reduction procedures for chronic obstructive pulmonary disease. Cochrane Database Syst Rev 2017; 2(doi):CD012158.
182. van der Ploeg JM, van der Steen A, Zwolsman S, van der Vaart CH, Roovers J. Prolapse surgery with or without incontinence procedure: a systematic review and meta-analysis. BJOG 2017
183. Veldman HD, Heyligers IC, Grimm B, Boymans TA. Cemented versus cementless hemiarthroplasty for a displaced fracture of the femoral neck: a systematic review and meta-analysis of current generation hip stems. Bone Joint J 2017; 99-B(4):421-31.
184. Vellayappan BA, Soon YY, Ku GY, Leong CN, Lu JJ, Tey JC. Chemoradiotherapy versus chemoradiotherapy plus surgery for esophageal cancer. Cochrane Database Syst Rev 2017; 8(doi):CD010511.
185. Vidale S, Agostoni E. Endovascular Treatment of Ischemic Stroke: An Updated Meta-Analysis of Efficacy and Safety. Vasc Endovascular Surg 2017; 51(4):215-19.
186. Wang CH, Zhang SY, Jin XF. Complete revascularization versus culprit-only revascularization in ST-segment elevation myocardial infarction and multivessel disease patients undergoing primary percutaneous coronary intervention: A meta-analysis and trial sequential analysis. International Journal of Cardiology 2017; 228(7):844-52.
187. Wang H, Man L, Li G, Huang G, Liu N, Wang J. Meta-Analysis of Stenting versus Non-Stenting for the Treatment of Ureteral Stones. PLoS One 2017; 12(1):e0167670.
188. Wang WW, Dong BC. Comparison on effectiveness of trans-septal suturing versus nasal packing after septoplasty: a systematic review and meta-analysis. Eur Arch Otorhinolaryngol 2017; 274(11):3915-25.
189. Wang X, He JJ, Chen X, Yang QQ. Stenting as a bridge to resection versus emergency surgery for left-sided colorectal cancer with malignant obstruction: A systematic review and meta-analysis. International Journal of Surgery 2017; 48(suppl_3):64-68.
190. Wang XC, Zhang D, Yang ZX, Gan JX, Yin LN. Mesh reinforcement for the prevention of incisional hernia formation: a systematic review and meta-analysis of randomized controlled trials. J Surg Res 2017; 209(doi):17-29.
191. Wang Y, Wen M, Zhou J, Chen Y, Zhang Q. Coronary artery bypass grafting versus percutaneous coronary intervention in patients with noninsulin treated type 2 diabetes mellitus: A meta-analysis of randomized controlled trials. Diabetes Metab Res Rev 2017;34(1):327-44.
192. Woltz S, Krijnen P, Schipper IB. Plate Fixation Versus Nonoperative Treatment for Displaced Midshaft Clavicular Fractures: A Meta-Analysis of Randomized Controlled Trials. J Bone Joint Surg Am 2017;9 9(12):1051-57.
193. Wu X, Liu Q, Zhang R, Wang W, Gao Y. Therapeutic efficacy and safety of laparoscopic surgery versus microsurgery for varicocele of adult males: A meta-analysis. Medicine (Baltimore) 2017; 96(34):e7818.
194. Xie L, Zhao ZG, Zhang SJ, Hu YB. Percutaneous vertebroplasty versus conservative treatment for osteoporotic vertebral compression fractures: An updated meta-analysis of prospective randomized controlled trials. Int J Surg 2017; 47(3):25-32.
195. Xu XL, Liu XD, Liang M, Luo BM. Radiofrequency Ablation versus Hepatic Resection for Small Hepatocellular Carcinoma: Systematic Review of Randomized Controlled Trials with Meta-Analysis and Trial Sequential Analysis. Radiology 2017; 287(2):461-72.
196. Yang L, Zhang B, Xing G, Du J, Yang B, Yuan Q, et al. Neoadjuvant chemotherapy versus primary debulking surgery in advanced epithelial ovarian cancer: A meta-analysis of peri-operative outcome. PLoS One 2017; 12(10):e0186725.
197. Yang S, Chen C, Wang H, Wu Z, Liu L. A systematic review of unilateral versus bilateral percutaneous vertebroplasty/percutaneous kyphoplasty for osteoporotic vertebral compression fractures. Acta Orthop Traumatol Turc 2017; 51(4):290-97.
198. Ye F, Zeng Z, Wang J, Liu H, Wang H, Zheng Z. Comparison of the use of rhBMP-7 versus iliac crest autograft in single-level lumbar fusion: a meta-analysis of randomized controlled trials. J Bone Miner Metab 2017; 36(1):119-27.
199. Zhang LL, Zhang Y, Ma X, Liu Y. Multiple cannulated screws vs. dynamic hip screws for femoral neck fractures: A meta-analysis. Orthopade 2017; 46(11):954-62.
200. Zhang S, Lan Z, Zhang J, Chen Y, Xu Q, Jiang Q, et al. Duct-to-mucosa versus invagination pancreaticojejunostomy after pancreaticoduodenectomy: a meta-analysis. Oncotarget 2017; 8(28):46449-60.
201. Zhang W, Li G, Chen YL. Should T-Tube Drainage be performed for choledocholithiasis after laparoscopic common bile duct exploration? a systematic review and meta-analysis of randomized controlled trials. Surg Laparosc Endosc Percutan Tech 2017:415–23.
202. Zhao X, Cui N, Wang X, Cui Y. Surgical strategies in the treatment of chronic pancreatitis: An updated systematic review and meta-analysis of randomized controlled trials. Medicine (Baltimore) 2017; 96(9):e6220.
203. Zhao XW, Ma JX, Ma XL, Li F, He WW, Jiang X, et al. Interspinous process devices(IPD) alone versus decompression surgery for lumbar spinal stenosis(LSS): A systematic review and meta-analysis of randomized controlled trials. Int J Surg 2017; 39(doi):57-64.
204. Zhu Q, Xu X, Yang X, Chen X, Wang L, Liu C, et al. Intramedullary nails versus sliding hip screws for AO/OTA 31-A2 trochanteric fractures in adults: A meta-analysis. Int J Surg 2017; 43(8):67-74.
